# Functional Characterization of Sesquiterpene Synthase from *Polygonum minus*

**DOI:** 10.1155/2014/840592

**Published:** 2014-02-11

**Authors:** Su-Fang Ee, Zeti-Azura Mohamed-Hussein, Roohaida Othman, Noor Azmi Shaharuddin, Ismanizan Ismail, Zamri Zainal

**Affiliations:** ^1^School of Biosciences and Biotechnology, Faculty of Science and Technology, Universiti Kebangsaan Malaysia, 43600 Bangi, Selangor, Malaysia; ^2^Institute of Systems Biology (INBIOSIS), Universiti Kebangsaan Malaysia, 43600 Bangi, Selangor, Malaysia; ^3^Faculty of Biotechnology and Biomolecular Sciences, Universiti Putra Malaysia, 43400 Serdang, Selangor, Malaysia

## Abstract

*Polygonum minus* is an aromatic plant, which contains high abundance of terpenoids, especially the sesquiterpenes C_15_H_24_. Sesquiterpenes were believed to contribute to the many useful biological properties in plants. This study aimed to functionally characterize a full length sesquiterpene synthase gene from *P. minus*. *P. minus* sesquiterpene synthase (*PmSTS*) has a complete open reading frame (ORF) of 1689 base pairs encoding a 562 amino acid protein. Similar to other sesquiterpene synthases, PmSTS has two large domains: the N-terminal domain and the C-terminal metal-binding domain. It also consists of three conserved motifs: the DDXXD, NSE/DTE, and RXR. A three-dimensional protein model for PmSTS built clearly distinguished the two main domains, where conserved motifs were highlighted. We also constructed a phylogenetic tree, which showed that PmSTS belongs to the angiosperm sesquiterpene synthase subfamily Tps-a. To examine the function of *PmSTS*, we expressed this gene in *Arabidopsis thaliana*. Two transgenic lines, designated as *OE3* and *OE7*, were further characterized, both molecularly and functionally. The transgenic plants demonstrated smaller basal rosette leaves, shorter and fewer flowering stems, and fewer seeds compared to wild type plants. Gas chromatography-mass spectrometry analysis of the transgenic plants showed that PmSTS was responsible for the production of **β**-sesquiphellandrene.

## 1. Introduction

Plants have developed a range of strategies to survive and adapt to their environment. One such strategy is to produce a large variety of secondary metabolites [1]. To date, there are an estimated 200,000 secondary metabolites that are produced by plants [2]. *Polygonum minus*, an aromatic plant that is indigenous to Malaysia, produces large number of secondary metabolites. Traditionally, *P. minus* is used to treat indigestion and dandruff problems and as a postnatal tonic [3]. This plant has a unique sweet and pleasant flavour and aroma and thus is commonly used in local cuisine. Its unique flavour is mainly due to the secondary metabolites present in the plant [4]. The secondary metabolites present in *P. minus* are also responsible for its useful biological properties, such as its antioxidant, antiulcer, antiviral, antimicrobial and antifungal activities [5, 6].

Secondary metabolites are divided into three major groups: terpenoids, alkaloids, and phenylpropanoids [7]. Terpenoids are the largest and most structurally diverse class. Different terpenoids are distributed unevenly within the plant kingdom [8]. Some terpenoids are restricted to one species or one genus [8]. The essential oils of *P. minus* have been shown to contain a high abundance of terpenoids especially the sesquiterpenes [4]. Twenty-four different types of sesquiterpenes including *α*-humulene, *α*-farnesene, *β*-farnesene, valencene, *α*-panasinsene, *α*-bergamotene, *β*-caryophyllene, *δ*-cadinene, and *α*-curcumene, have been identified, to date [4]. This diversity suggests that *P. minus* is a good source of sesquiterpenes for research on secondary metabolites, particularly the sesquiterpenes. Previously, most studies focused on a metabolomic approach to examine this plant, whereas those adopting a molecular approach for characterizing and understanding the biosynthetic regulation of sesquiterpene related genes are still lacking.

Based on our previously constructed cDNA-AFLP transcriptome profiles, a sesquiterpene synthase gene (GenBank: HO079100 and HO079108) was shown to be highly upregulated upon stress induced by salicylic acid [9]. Although transcriptomic data are available, the major challenge for studies of *P. minus* is the lack of a transformation and regeneration system for the functional study of its genes. To address this concern, the model plant *A. thaliana* was used in this work. *A. thaliana* is the first angiosperm to have had its complete genome sequenced. Since then, it has been well studied. Its short life cycle of only 3 months and its ability to produce a large number of progeny seeds have encouraged many researchers to use *A. thaliana *for functional studies [10]. The well-established floral-dip transformation system also provides a fast and efficient method to transfer genes from *Agrobacterium* into *A. thaliana* [11]. This method involves only a simple immersion of the floral buds in an *Agrobacterium* suspension.

*PmSTS* was previously cloned and expressed in *Lactococcus lactis* [12]. That study was aimed solely at maximizing metabolite production in a bacterial system and did not study how the gene is regulated in plants. In addition, the *PmSTS* gene introduced into *L. lactis* contained two incorrect nucleotides that resulted in a single amino acid change [12], calling into question the true function of this protein.

In this study, we expressed the correct sequence of *PmSTS *to determine the function of its protein product in a model plant system. As there is no established transformation and regeneration system available in *P. minus*, the model plant *A. thaliana* was used to investigate the role of *PmSTS* in plants. The overexpression of *PmSTS* in *A. thaliana* provides a better understanding of the gene function. The results of this study further enhance our understanding of the biosynthesis and regulation of secondary metabolites in *P. minus*, particularly the sesquiterpenes.

## 2. Materials and Methods

### 2.1. Plant Material and Growth Conditions

*A. thaliana* ecotype Columbia-0 was grown in a growth chamber (Conviron) at a temperature of 22°C day/20°C night and relative humidity of 50–70%. The photoperiod was set at 16 h day/8 h night, with a light intensity of 100–150 *μ*moles m^−2^ s^−1^ using fluorescent bulbs. The plants were ready for floral-dip transformation one week after the primary inflorescences were clipped. Watering was stopped three days prior to transformation to increase the transformation efficiency.

### 2.2. In Silico Analysis of *PmSTS*

The nucleotide sequence of *PmSTS* was retrieved from the NCBI database with GenBank ID of JX025008. The physiochemical properties of the PmSTS were determined using PROTPARAM software (http://web.expasy.org/protparam/). The presence of signal peptide was predicted using SignalP 4.1 software (http://www.cbs.dtu.dk/services/SignalP/) [13]. Comparative sequence analysis of *PmSTS* was performed using NCBI BLAST against the protein database (http://blast.ncbi.nlm.nih.gov/). Multiple sequence alignment was done with BIOEDIT software using the default parameters (http://www.mbio.ncsu.edu/bioedit/bioedit.html). The three-dimensional (3D) protein structure homology-modelling of the PmSTS was generated using I-TASSER software [14]. The stereochemical quality of the predicted 3D protein structure was examined through PROCHECK analysis (http://www.ebi.ac.uk/thornton-srv/software/PROCHECK/). Phylogenetic tree was built using MEGA5 software with neighbour joining method. Bootstrap of 1000 replicates was done. Terpene synthases from seven previously recognized TPS subfamilies Tps-a to Tps-g were retrieved from the NCBI GenBank database according to Bohlmann et al. [15] and Danner et al. [16]. The Tps-c and Tps-e subfamilies, which are composed of the copalyl diphosphate (cdp) synthases and kaurene synthases and are involved in primary metabolism, were chosen as outgroups.

### 2.3. Gene Amplification and Construction of the pCAMSS overexpression Vector

*PmSTS *(GenBank: JX025008) was amplified by standard PCR methods using the *PmSTS* specific forward primer 5′-GGGCAGATCTT**ATG**TATTCCATGATC-3′ and reverse primer 5′-GGCTGGTGACC **TTA**TATCAGTATGGG-3′. To facilitate the cloning process, restriction enzymes (RE) sites for *Bgl*II and *BstE*II (underlined) were attached to the 5′ ends of the forward and reverse primers, respectively. The nucleotides in bold type are the start (**ATG**) and termination (**TTA**) codons in the open reading frame of the *PmSTS* gene.

**Figure 1:**
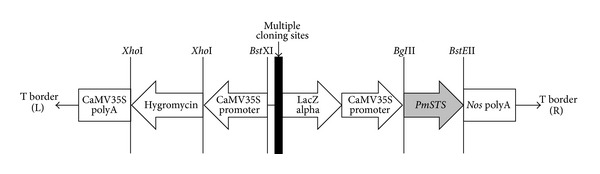
Schematic diagram of the T-DNA region of pCAMSS vector. The pCAMSS recombinant vector contains the full length *PmSTS* gene, which is expressed under the control of the constitutive CaMV35S promoter.

The vector pCAMBIA1301 (Centre for the Application of Molecular Biology of International Agriculture, Black Mountain, Australia) was used as the backbone for the construction of the plant transformation vector, pCAMSS, which harboured the *PmSTS* gene. To construct the pCAMSS vector, the *β*-glucuronidase (GUS) reporter gene was first excised from the pCAMBIA1301 vector and then replaced with the PCR-amplified *PmSTS* gene. Both the pCAMBIA1301 vector and the *PmSTS* gene were digested with the *Bgl*II and *BstE*II restriction enzymes to generate complementary sticky ends for ligation. The digested fragment of the *PmSTS* gene was ligated into the corresponding sites of pCAMBIA1301, yielding the pCAMSS vector (Figure 1). The pCAMSS vector was then transformed into *Agrobacterium tumefaciens* strain GV3101. The cloned pCAMSS vector was sent for sequencing and RE digestion to confirm the integration of the *PmSTS* gene in the correct orientation.

### 2.4. *Agrobacterium*-Mediated Floral-Dip Plant Transformation

*A. thaliana *was transformed using the *Agrobacterium*-mediated floral dip method [11]. *Agrobacterium* cells were grown to an OD_600_ of 0.7. The floral-dip inoculation medium contained harvested cells that were resuspended in 5% sucrose and 0.05% Silwet. The secondary inflorescences were immersed in the inoculation medium and swirled gently to allow the intake of *Agrobacterium* harbouring the pCAMSS vector into the flower gynoecium. The transformed plants were kept in the dark and wrapped with plastic overnight to maintain humidity. The next day, the plants were returned to their normal growth conditions. The transformation was repeated after a week to increase the transformation efficiency. Plants were grown for additional 4-5 weeks, until all of the siliques became brown and dry. The seeds were harvested and stored at 4°C under desiccation.

### 2.5. Selection of Transgenic *A. thaliana*

Seeds were surface sterilized with 50% Clorox containing 0.05% Tween-20 for 10 min, followed by 80% ethanol for 2 min, and the seeds were then rinsed three times with distilled water before plating. To select the transformed plants, approximately 100 sterilized seeds were screened on Murashige and Skoog (MS) solid media containing 25 mg L^−1^ hygromycin. The seeds were cultivated following the standard method of Harrison et al. [17]. The plated seeds were stratified at 4°C for 2 days. The seeds were then placed under light for 6 h to induce germination, followed by 2 days of incubation in the dark, and then returned to normal growth conditions. The putative transgenic plants were selected by two weeks of growth on hygromycin plates. The putative transformants grown on selection media had long hypocotyls, green leaves and long roots. These putative transformants were transferred to pots with soil and grown under normal growth conditions. The seeds from the mature plants were harvested after one month.

To verify the presence of the *PmSTS* gene, DNA from the putative transformants and wild type *A. thaliana* was extracted using the standard CTAB extraction method [18]. Wild type *A. thaliana* were used as the negative control for the PCR amplification. Genomic PCR was performed using a forward primer containing a region of the CaMV35S promoter (5′-TCCCACTATCCTTCGCAAGACCC-3′) and a reverse primer containing a *PmSTS* gene-specific sequence (5′-AGTGATAGGCAACTCCAAGC-3′).

### 2.6. Semiquantitative RT-PCR Analysis of the Transgenic *A. thaliana*

Semiquantitative RT-PCR was conducted to compare the expression of the *PmSTS* gene in the T_2_ transgenic *A. thaliana* and wild type *A. thaliana*. Total RNA was extracted from the leaves using the TRI Reagent (Molecular Research Centre, Inc. Cincinnati, OH, USA), according to the manufacturer's instructions. First strand cDNA was synthesized with the Maxima First Strand cDNA Synthesis Kit (Thermo Fisher Scientific, USA) using total RNA as the template. Semiquantitative RT-PCR analysis was performed using standard PCR methods with 500 ng of cDNA template. The forward primer (5′-CCATGATGCAGCCAACCGAGAT-3′) corresponded to nucleotide 1523–1544 of the *PmSTS* gene, while the reverse primer (5′-AATCCATCCTCTCCGGCGTCAT-3′) corresponded to nucleotide 1622–1643. As a control, a PCR with the housekeeping gene 4HPPD (GenBank: AT1G06570.1/NM 100536) was performed in parallel using 5′-GCGCTTCCATCACATCGAGTTC-3′ and 5′-AATCCAATGGGAACGACGACGC-3′ as the forward and reverse primers, respectively.

### 2.7. GC-MS Analysis of The Transgenic *A. thaliana*

The volatiles emitted from the leaf samples were extracted using the headspace solid-phase microextraction (HS-SPME) method. A polydimethylsiloxane-(PDMS-) coated fibre was exposed to the headspace of the sample vial containing 2 g of leaves for 30 min at 55–60°C before injection into the gas chromatograph mass spectrometer (GC-MS). The GC-MS analysis was performed on an Agilent 7890A gas chromatograph (GC) that was directly coupled to the mass spectrometer system (MS) of an Agilent 5975C inert MSD with a triple-axis detector. Separation was achieved with a 5% phenyl methylpolysiloxane column (model AB-5MS; Abel Industries) that was 30 m long and 0.25 mm in diameter and had a film thickness of 0.25 *μ*m. Helium was used as the carrier gas, with a flow rate of 1.3 mL min^−1^. A splitless injection was set at 50°C hold for 3 min, increased to 250°C at a rate of 6°C min^−1^, and hold at 250°C for 5 min. The peaks were identified by searching the NIST/EPA/NIH mass spectral library (version 2.0), and the results were combined in a GC-MS chromatogram.

## 3. Results and Discussion

Sesquiterpene synthases broadly refer to enzymes that convert farnesyl diphosphate (FPP) into various sesquiterpenes. Previous studies of sesquiterpenes often comment on the structural complexity and diversity of sesquiterpene metabolism. The main causes of sesquiterpene diversity are the large number of different sesquiterpene synthases that are expressed in plants and the ability of some sesquiterpene synthases to form multiple products from a single FPP substrate [19]. The *PmSTS* gene has an open reading frame of 1689 bp, which encodes a 562 amino acid protein with a calculated molecular mass of 65.1 kDa and a theoretical isoelectric point (pI) of 5.29. The deduced amino acid sequence of PmSTS showed no signal peptide, which is consistent with other sesquiterpene synthases of 550–580 amino acids, and is shorter than monoterpene synthases (600–650 amino acids). The absence of an N-terminal plastid targeting signal peptide suggests that PmSTS is localized to the cytosol where FPP is found and where sesquiterpene biosynthesis takes place.

**Figure 2:**
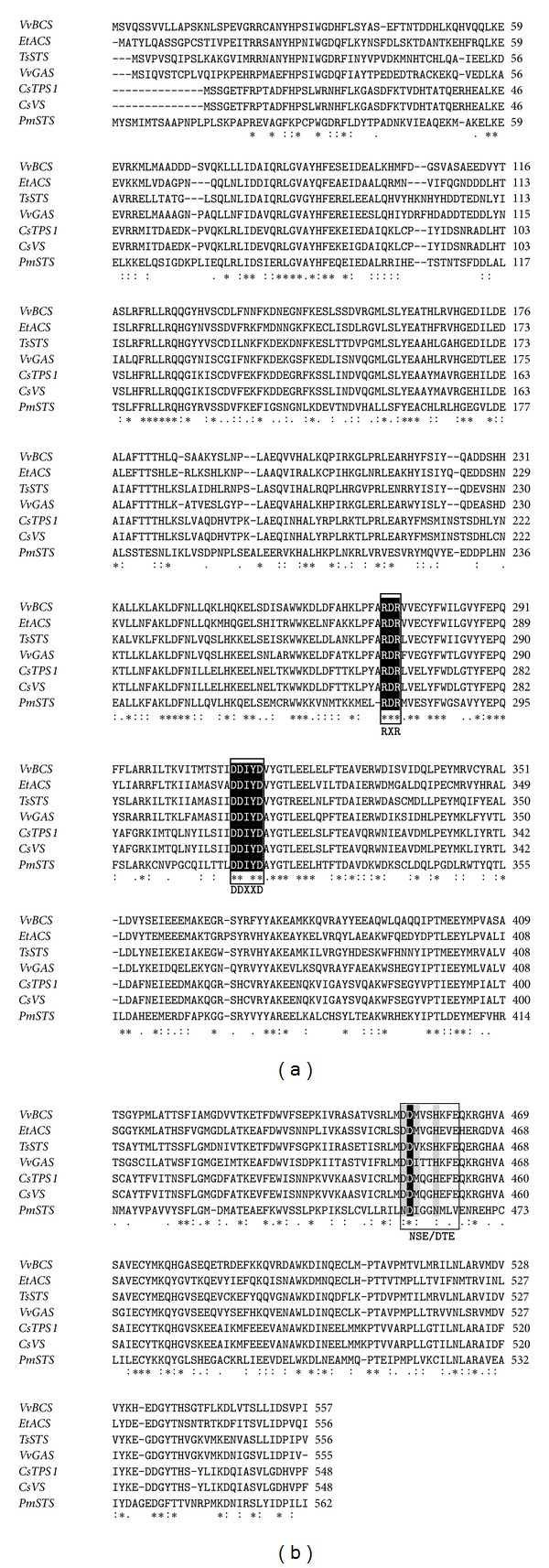
Multiple sequence alignment. The deduced amino acid sequence of *PmSTS* was aligned with homologues identified from the BLASTX analysis. Sequences highlighted in black indicate identical residues, while those in grey indicate similar residues. The conserved motifs RXR, DDXXD, and NSE/DTE are marked. *PmSTS*: sesquiterpene synthase [*P. minus*]; *TsSTS*: sesquiterpene synthase [*Toona sinensis*]; *CsTPS1*: terpene synthase 1 [*Citrus sinensis*]; *CsVS*: valencene synthase [*Citrus sinensis*]; *VvGAS*: germacrene A synthase [*Vitis vinifera*]; *VvBCS*: (E-) beta-caryophyllene synthase [*Vitis vinifera*]; *EtACS*: alpha-copaene synthase [*Eleutherococcus trifoliatus*].

Based on the BLASTX analysis (Table 1), the closest homologue to PmSTS is the sesquiterpene synthase from *Toona sinensis*, with which it shares 43% identity. Although the level of amino acid sequence similarity between PmSTS and the other homologues was relatively low (≤43%), multiple sequence alignment identified several conserved motifs that are found in typical terpene synthases (Figure 2). The two highly conserved aspartate-rich motifs DDXXD (residues 314–318) and NSE/DTE (residues 465–473), which are found in most of the sesquiterpene synthases were highlighted in Figure 2, together with the other commonly conserved RXR motif (residues 277–279) region. The DDXXD and NSE/DTE motifs have been reported to flank the entrance of the active site [20]. They are involved in binding a trinuclear magnesium cluster, with DDXXD binding two magnesium ions and NSE/DTE binding one magnesium ion [21]. Catalysis of the FPP substrate occurs when it reaches the hydrophobic active site, where the diphosphate moiety of FPP interacts with the magnesium ions [22]. Thus, this magnesium cluster is important for the positioning of FPP in the hydrophobic substrate binding pocket of PmSTS [23].

**Figure 3:**
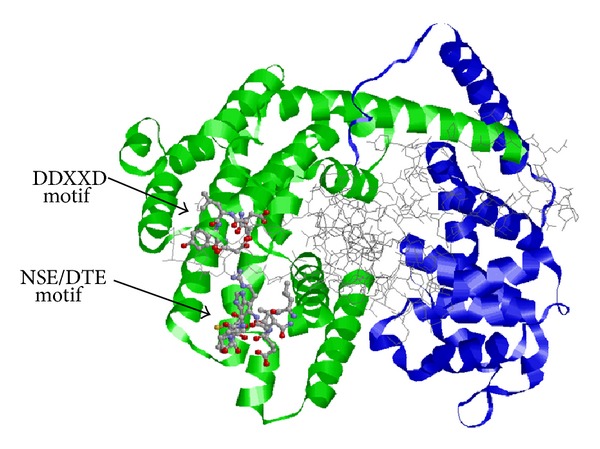
Predicted 3D model of PmSTS generated by the I-TASSER server. The two conserved domains are highlighted. The N-terminal domain is in blue and the C-terminal domain is in green. Both aspartate-rich motifs are displayed using ball and stick representation with CPK colour. The wire bonds represent the rest of the protein.

**Figure 4:**
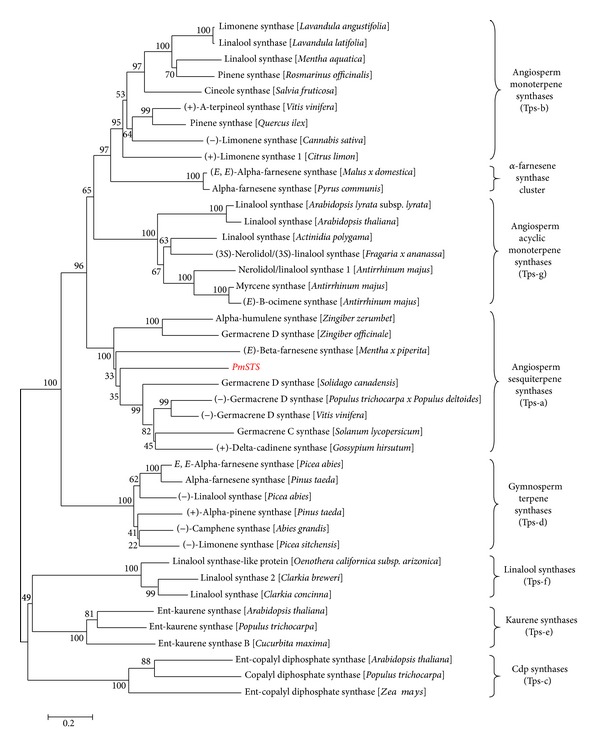
Phylogenetic tree of PmSTS with selected terpene synthases from other plants. Seven previously identified TPS subfamilies (Tps-a to Tps-g) were chosen based on Bohlmann et al. [15] and Danner et al. [16]. The Tps-c and Tps-e subfamilies, which are composed of the copalyl diphosphate (cdp) synthases and kaurene synthases and are involved in primary metabolism, were chosen as outgroups. The alignment was performed using the Clustal Omega algorithm. The tree was built using the neighbour joining method and 1000 replicates for bootstrapping. The numbers indicated are the actual bootstrap values of the branches.

Similar to other sesquiterpene synthases, PmSTS contains two large conserved domains, which were identified in a PFAM search; PF01397 corresponds to the terpene synthase family N-terminal domain, and PF03936 corresponds to the terpene synthase family C-terminal metal-binding domain. These domains are shown in the 3D model built using I-TASSER in Figure 3. The 3D protein model was constructed using 5-epi-aristolochene synthase (TEAS) [PDB accession: 1HXG] as a template. The quality of the PmSTS 3D protein model was checked using a Ramachandran plot analysis [24]. The PmSTS 3D protein model exhibited a good fit with the reference geometry with 90.4% of nonglycine and nonproline residues in the most favoured regions. From the 3D model, PmSTS was shown to consist entirely of *α*-helices and short connecting loops and turns. Both of the conserved aspartate-rich regions, the DDXXD, and NSE/DTE motifs were found in the C-terminal domain demonstrating the importance of the C-terminal domain, which contains the active site, for the catalysis of the substrate FPP [25]. While the actual function of the N-terminal domain remains unknown, it has been suggested to be involved in facilitating the proper folding of the catalytically active C-terminal domain [26]. Phylogenetic analysis of the deduced amino acid sequence of PmSTS showed that it belongs to the Tps-a subfamily of angiosperm sesquiterpene synthases (Figure 4) [15, 16].

**Figure 5:**
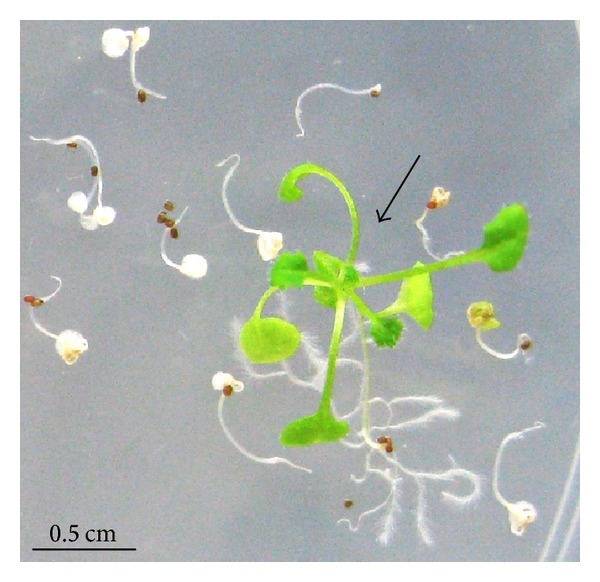
Comparison of hygromycin-sensitive and hygromycin-resistant (shown in arrow) seedlings. The seedlings were screened for 20 days on selection plate containing MS media supplemented with 25 mg L^−1^ hygromycin.

The role and product specificity of PmSTS were determined by generating transgenic *A. thaliana*. Overexpression of PmSTS in *A. thaliana* was accomplished using *Agrobacterium* harbouring the transformation vector pCAMSS. Using the *Agrobacterium*-mediated floral-dip transformation method, ten hygromycin-resistant transgenic *A. thaliana* were successfully generated. These plants had long hypocotyls, green leaves, and long main roots with the formation of lateral roots (Figure 5). In contrast, the nontransformants showed short hypocotyls, bleached out leaves, and no lateral root formation (Figure 5). The putative transformants were further verified using PCR amplification of the plant genomic DNA. Fully mature leaves from ten 60-day-old putative transgenic plants and one wild type plant were collected for DNA extraction. All the putative transformants gave rise to a band of the expected size of 365 bp, while the wild type plant showed no amplification. This result confirmed the presence of the *PmSTS* gene in the genomes of the transgenic plants.

**Figure 6:**
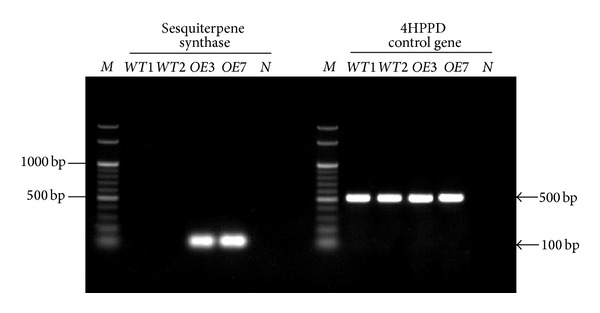
Semiquantitative RT-PCR for sesquiterpene synthase (*PmSTS*). PCR for the 4HPPD gene was performed in parallel as a positive control. M: 100 bp DNA ladder; *WT*1 and *WT*2: wild type *A. thaliana* plants; *OE*3: *OE*3 transgenic plant; *OE*7: *OE*7 transgenic plant; *N*: no template control.

**Figure 7:**
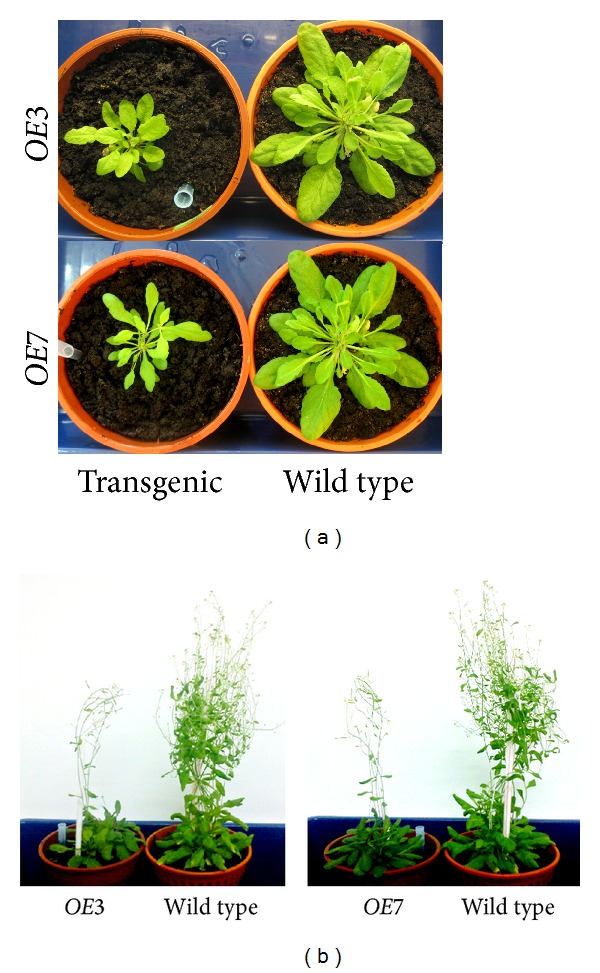
Comparison of the phenotypes of the transgenic *A. thaliana* and wild type *A. thaliana*. (a) Plants (2-month-old) were grown on soil for 1 month. Upper panel: *OE*3 transgenic plant and wild type plant. Lower panel: *OE*7 transgenic plant and wild type plant. (b) Two 4-month-old *OE*3 and *OE*7 T_2_ plants compared with a 3-month-old wild type plant.

Two of the transgenic plants, designated as *OE*3 and *OE*7, were selected for further analysis. Semiquantitative RT-PCR analysis was performed. Both the *OE*3 and *OE*7 plants showed high expression of the *PmSTS *gene by the amplification of a distinct band at 100 bp, which was absent in the wild type plants (Figure 6). Meanwhile, from the morphological analysis, both plants showed delayed growth compared to the wild type plants (Figure 7). The *OE*3 and *OE*7 plants took an additional month to reach the seed maturation step. This phenotype was inheritable, as the T_2_ plants of these two lines also showed similar growth retardation. The plants also demonstrated smaller basal rosette leaves and shorter and fewer flowering stems. Although flowers and viable seeds were still produced from these plants, the number of seeds obtained was halved compared to the wild type plants.

**Figure 8:**
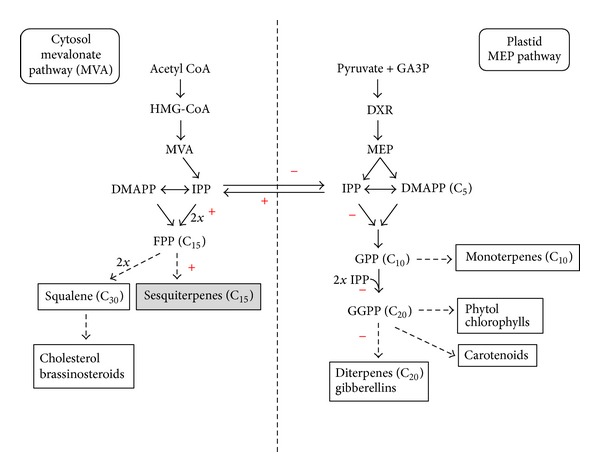
A simplified version of the sesquiterpene biosynthetic pathway. This pathway shows the predicted flux of substrate (IPP and FPP) to produce more sesquiterpenes (C_15_) in transgenic plants overexpressing the *PmSTS *gene. (+) indicates the increased flux of substrate and (−) indicates the decreased flux of substrate. GA3P: glyceraldehyde 3-phosphate. HMG-CoA: 3-hydroxy-3-methylglutaryl-CoA. DXR: 1-deoxy-D-xylulose 5-phosphate reductoisomerase. MVA: mevalonate acid. MEP: methyl erythritol phosphate. IPP: isopentyl pyrophosphate and its isomer DMAPP: dimethylallyl diphosphate. FPP: farnesyl diphosphate. GGPP: geranylgeranyl diphosphate (modified from Okada et al. [31] and Vickers et al. [32]).

It has been suggested that the overexpression of the *PmSTS* gene in transgenic plants interferes with the IPP substrate pool in the cytosol (Figure 8). This overexpression causes the channelling of more isopentyl pyrophosphate (IPP), the building block for terpenes, and FPP to the overexpressed PmSTS. This certainly lowers the flux of IPP to the plastids for the synthesis of other essential and larger isoprene products that are important for the plant growth, such as gibberellins (GA) [27, 28]. This model was further supported when many of the gene modifications involving terpenoid biosynthesis, such as the overexpression of the strawberry linalool/nerolidol synthase (monoterpene) and taxadiene synthase in *A. thaliana*, also resulted in a dwarf phenotype due to a decrease in the level of GA [27, 29]. The GA-deficient *A. thaliana* mutants designated as *dwarf and delayed-flowering 1* (*ddf1*) also demonstrated a similar reduction in plant size and other similar phenotypes [30].

**Figure 9:**
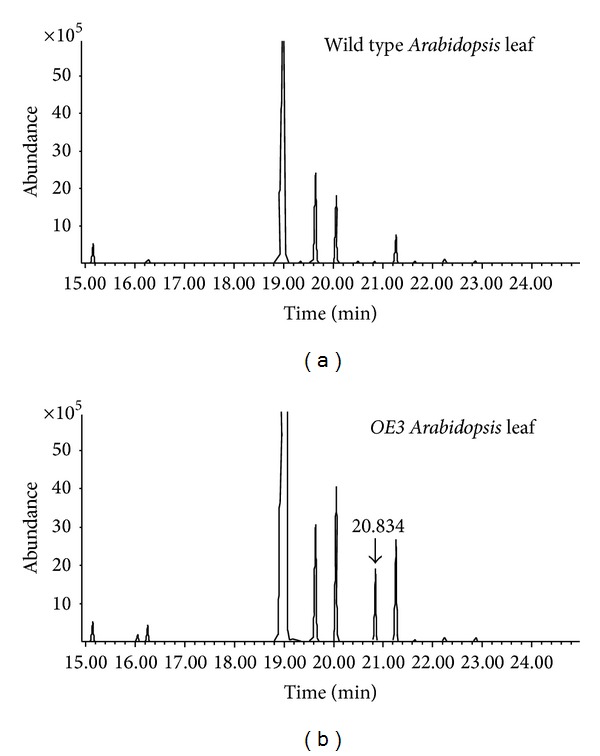
GC-MS chromatograms of the transgenic *OE*3 (b) and a wild type plant (a). The arrow (↓) indicates the peak at the retention time of 20.834 that shows high match with *β*-sesquiphellandrene in the NIST/EPA/NIH library.

**Figure 10:**
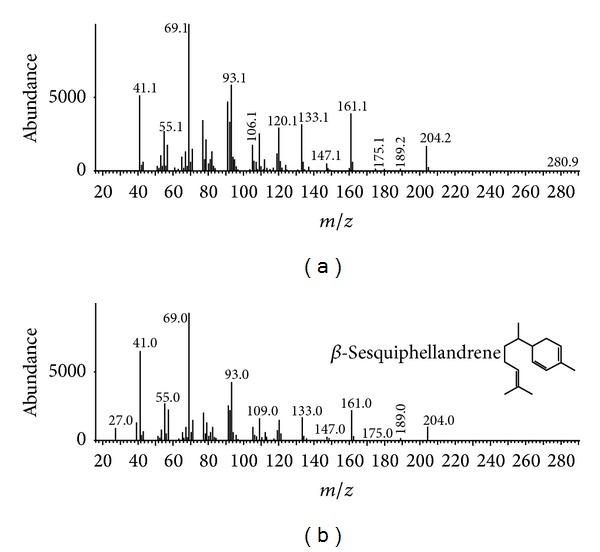
Mass spectra of *β*-sesquiphellandrene. (a) shows the mass spectrum for the sample compound, while (b) shows the mass spectrum of the highest hit in the NIST/EPA/NIH library.

A GC-MS analysis was performed to identify the specific product produced by transformation with the *PmSTS* gene. In this analysis, material extracted from the *A. thaliana* leaf samples was examined using the HS-SPME method. By using leaf samples, we were able to reduce the detection of background terpenes, as *A. thaliana* leaves were previously reported to not emit or to only emit traces of terpene volatiles [33]. In addition, GC-MS analysis was performed with wild type plants as a control. The GC-MS analysis yielded two chromatograms with similar patterns (Figure 9). However, a very clear difference was observed for the transgenic plant *OE*3, as an additional peak was present at the retention time of 20.834. This peak was identified as *β*-sesquiphellandrene, based on the closest hit from a search of the NIST/EPA/NIH library (version 2.0). The mass spectrum of the *β*-sesquiphellandrene peak compared with that of the highest hit from the library was shown in Figure 10. The production of *β*-sesquiphellandrene by PmSTS was in agreement with the findings from Song et al. [12]. This result also showed that the point mutation introduced by Song et al. [12] at K266E does not affect the product specificity of PmSTS in producing *β*-sesquiphellandrene. Having obtained the same product in both *L. lactis* and *A. thaliana*, we have showed that PmSTS was responsible for the production of *β*-sesquiphellandrene through the common mevalonate pathway in sesquiterpene biosynthesis.

## 4. Conclusion

The diversity of the sesquiterpenes found in *P. minus* renders this plant a major resource for research related to sesquiterpene biosynthesis. In this study, we applied transgenic technology by overexpressing *PmSTS* in *A. thaliana*. Growth retardation was clearly observed in the transgenic lines *OE*3 and *OE*7. These two plants showed a high expression of the *PmSTS* gene, which resulted in the production of *β*-sesquiphellandrene. The *β*-sesquiphellandrene produced in these *A. thaliana* transgenic plants indicated the effectiveness of *A. thaliana* in synthesizing the same product as in previously expressed *L. lactis* through the common mevalonate pathway of sesquiterpene biosynthesis. This research strongly suggests the potential of the *A. thaliana* plant system for the study of the sesquiterpene synthase genes in *P. minus*.

## Figures and Tables

**Table 1:** BLASTX analysis. *PmSTS* was compared with the NCBI protein database for gene identification purposes.

Description^a^	Organism	*E* value	Identity (%)
Sesquiterpene synthase	*Toona sinensis *	5*e* ^−147^	43
Terpene synthase 1	*Citrus sinensis *	2*e* ^−135^	42
Valencene synthase	*Citrus sinensis *	2*e* ^−133^	42
Germacrene A synthase	*Vitis vinifera *	4*e* ^−141^	42
(*E*)-Beta-caryophyllene synthase	*Vitis vinifera *	2*e* ^−136^	42
Alpha-copaene synthase	*Eleutherococcus trifoliatus *	2*e* ^−136^	42
Germacrene D synthase	*Actinidia deliciosa *	6*e* ^−135^	41
Germacrene B synthase	*Cistus creticus *subsp. *creticus *	8*e* ^−130^	41
(+)-Delta-cadinene synthase	*Gossypium arboreum *	4*e* ^−136^	41
Terpene synthase	*Camellia sinensis *	6*e* ^−137^	41
Beta-curcumene synthase	*Vitis vinifera *	2*e* ^−130^	41

^a^Description—homology search using BLASTX.
